# Association of Triglyceride–Glucose Index and Coronary Chronic Total Occlusion in Patients Undergoing Coronary Angiography: A Retrospective Study

**DOI:** 10.3390/jcdd13060275

**Published:** 2026-06-17

**Authors:** Yan Li, Puhan Song, Mengyi Zheng, Yanyao Jia, Juan Wang, Qian Zhang, Xiaorong Xu, Zhiyong Zhang, Zongsheng Guo, Lin Zhao, Jing Cheng

**Affiliations:** 1 Department of Cardiology, Beijing Chaoyang Hospital, Capital Medical University, Beijing 100020, China; ly18811125001@163.com (Y.L.); 18331341285@163.com (M.Z.); jiayanyao@126.com (Y.J.); wangjuan.goal@163.com (J.W.); zqian604@163.com (Q.Z.); medicinexxr@163.com (X.X.); auysun@126.com (Z.Z.); guozongsheng@sohu.com (Z.G.); 2 Department of Pulmonary and Critical Care Medicine, Xuanwu Hospital, Capital Medical University, Beijing 100053, China; sph98176@163.com

**Keywords:** triglyceride–glucose index, insulin resistance, chronic total occlusion, coronary artery disease

## Abstract

Background: The triglyceride–glucose (TyG) index is a simple surrogate marker of insulin resistance (IR) and has been associated with coronary artery disease (CAD). However, the association between the TyG index and coronary chronic total occlusion (CTO) remains limited. Methods: In this retrospective study, 1157 patients who underwent coronary angiography at Beijing Chaoyang Hospital from January 2024 to January 2026 were enrolled and classified into the CTO group (n = 317) and the non-CTO group (n = 840). Multivariable logistic regression analyses were performed to assess the association between the TyG index and CTO. Restricted cubic spline analysis was used to examine the linear dose–response relationship. Subgroup analyses were conducted according to age, sex, smoking status, hypertension, and diabetes mellitus. Results: Patients with CTO had a significantly higher TyG index than those without CTO (8.98 [8.46, 9.45] vs. 8.79 [8.41, 9.26], *p* = 0.003). In the multivariable logistic regression analysis, the TyG index was independently associated with the presence of CTO (OR = 1.377, 95% CI 1.082–1.752, *p* = 0.009). In a sensitivity analysis further adjusted for diabetes mellitus, the association remained significant (OR = 1.356, 95% CI 1.052–1.747, *p* = 0.018). Restricted cubic spline curve analysis showed a nonlinear dose–response relationship (*p* for nonlinear = 0.005) between the TyG index and CTO risk. In the subgroup analyses, the association was directionally consistent across subgroups. Nominally significant associations were observed in elderly participants (OR 1.68, 95% CI 1.20–2.37, *p* = 0.003), men (OR 1.40, 95% CI 1.06–1.86, *p* = 0.018), and patients with hypertension (OR 1.55, 95% CI 1.14–2.11, *p* = 0.005). Conclusions: An elevated TyG index was independently associated with the presence of CTO. The association was generally consistent across major clinical subgroups, with no significant interactions observed.

## 1. Introduction

Insulin resistance (IR) is an important contributor to coronary artery disease (CAD) [1,2]. The hyperinsulinemic–euglycemic clamp is the gold standard method for assessing insulin sensitivity, but its complex experimental procedure has limited its widespread use in clinical practice [3]. The triglyceride–glucose (TyG) index, calculated from fasting glucose and triglyceride (TG) levels, is a simple, reliable, and economical indicator of IR, and it correlates well with the hyperinsulinemic–euglycemic clamp test [4,5,6]. Current evidence indicates that patients with an elevated TyG index have a higher risk of CAD and a worse prognosis [7,8,9,10,11]. A recent systematic review and meta-analysis further demonstrated that individuals with higher TyG index values had a greater risk of CAD, more severe coronary lesions, and a worse prognosis than those with lower TyG index values [7]. In addition, angiography-based studies have linked elevated TyG values to more complex coronary anatomy, multivessel disease, and greater atherosclerotic burden, particularly in patients with acute coronary syndrome or established CAD [8,11]. These findings suggest that the TyG index may be related to the severity and complexity of coronary atherosclerosis.

Coronary chronic total occlusion (CTO) is one of the most complex manifestations of CAD and is closely linked to ischemic burden, impaired ventricular function, persistent symptoms, challenging revascularization, and adverse clinical outcomes [12,13]. Recent consensus statements highlight the need for careful assessment of symptoms, ischemia, viability, ventricular function, lesion complexity, and expected revascularization benefit in patients with CTO [12]. Although CTO-PCI strategies continue to advance, patient evaluation and treatment selection remain challenging [12,14]. These considerations support the need to identify simple metabolic markers associated with the presence of CTO in patients undergoing coronary angiography.

Previous studies have shown that a higher TyG index is associated with less developed coronary collateralization and poorer clinical outcomes in patients with CTO [15,16,17]. Recently, Xiao et al. [18] provided direct evidence indicating that the TyG index is significantly associated with CTO in a single-center cross-sectional study involving 2691 patients, of whom 688 (25.6%) were identified as CTO patients, from southwest China. Although Xiao’s study provided significant initial evidence, direct investigation into the relationship between the TyG index and the presence of CTO remains limited, and additional validation in independent populations with different geographical and clinical characteristics is needed to clarify the generalizability and clinical relevance of this association. Moreover, most available studies have not fully addressed whether the association between the TyG and CTO is independent of diabetes status or whether it differs across clinically relevant subgroups. Therefore, this study aimed to evaluate the association between the TyG index and the presence of coronary CTO in an independent angiography-based cohort from northern China, with additional analyses accounting for clinically relevant subgroups, including diabetes status.

## 2. Methods

### 2.1. Study Population and Data Collection

We retrospectively screened patients who underwent diagnostic coronary angiography at the Department of Cardiology, Beijing Chaoyang Hospital, Capital Medical University, between January 2024 and January 2026 because of angina-like symptoms or suspected significant coronary stenosis on coronary computed tomography angiography. Demographic information, cardiovascular risk factors, laboratory measurements, transthoracic echocardiographic parameters, and angiographic findings were extracted from the electronic medical record system. Patients were excluded if they had a history of percutaneous coronary intervention (PCI) or coronary artery bypass grafting (CABG), evidence of acute infection or inflammatory disease, hematologic disorders, autoimmune disease, or incomplete key clinical or angiographic data. Acute infection or inflammatory disease was identified according to documented clinical diagnoses, symptoms, signs, and available laboratory findings during hospitalization. No single biomarker threshold, such as a predefined white blood cell count or C-reactive protein cut-off, was used because inflammatory biomarkers were not systematically measured in this retrospective cohort. Information on lipid-lowering and glucose-lowering medications before coronary angiography was not systematically available in the electronic medical records used for this retrospective analysis and, therefore, could not be included as covariates in the multivariable models. The study protocol conformed to the principles of the Declaration of Helsinki and was approved by the Ethics Committee of Beijing Chaoyang Hospital, Capital Medical University (Approval No. 2020-ke-2). The requirement for written informed consent was waived because of the retrospective design and the minimal risk to participants.

### 2.2. Calculation of the TyG Index

The TyG index was calculated as Ln [fasting triglyceride (mg/dL) × fasting glucose (mg/dL)/2] [4]. Venous blood samples were obtained from the antecubital vein after an overnight fast of at least 8 h and were analyzed in the hospital central laboratory using a Hitachi 711 Chemistry Analyzer under standardized quality-control procedures. Fasting plasma glucose was determined using the hexokinase method, and triglyceride concentrations were measured using an enzymatic colorimetric assay.

### 2.3. Definition of CTO

Coronary angiography was performed in all patients by experienced interventional cardiologists according to standard clinical practice. CTO was defined as complete coronary artery occlusion with Thrombolysis in Myocardial Infarction (TIMI) grade 0 flow and an estimated or documented duration of at least 3 months [19]. Patients were categorized into the CTO and non-CTO groups on the basis of coronary angiographic findings.

### 2.4. Statistical Analysis

Continuous variables are reported as the median and interquartile range (IQR), and between-group comparisons were performed using the Wilcoxon rank-sum test. For comparisons among TyG tertiles, the Kruskal–Wallis test was used for continuous variables. Categorical variables are presented as numbers and percentages and were compared using the chi-square test or Fisher’s exact test, as appropriate. Associations between the TyG index and the presence of CTO were evaluated using multivariable logistic regression models, with odds ratios (ORs) and 95% confidence intervals (CIs) reported. Candidate covariates included age, sex, current smoking, hypertension, creatinine, total cholesterol, LDL-C, HDL-C, hemoglobin, platelet count, and left ventricular ejection fraction (LVEF). Multivariable logistic regression was performed using the Forward: LR method, and the TyG index was entered either as a continuous variable or as tertiles. To address the potential confounding effect of diabetes mellitus, an additional sensitivity model was constructed by further adjusting for diabetes status. The discriminative performance of the TyG index for identifying CTO was assessed using receiver operating characteristic (ROC) curve analysis and the corresponding area under the curve (AUC). The dose–response relationship between the TyG index and CTO was explored using restricted cubic spline models, with the median TyG value set as the reference point. The spline model was adjusted for the variables retained in the final multivariable logistic model, including current smoking, LDL-C, HDL-C, hemoglobin, and LVEF. Subgroup analyses according to age (<60 or ≥60 years), sex (male or female), smoking (yes or no), hypertension (yes or no), and diabetes mellitus (yes or no) were carried out. The interaction between each subgroup variable and the TyG index was analyzed. Statistical analyses were performed using SPSS version 25.0 (SPSS, Chicago, IL, USA) and R software version 4.2.1 (R Foundation for Statistical Computing, Vienna, Austria). A two-sided *p* value < 0.05 was defined as the threshold of statistical significance.

## 3. Results

### 3.1. Baseline Characteristics

A total of 1157 patients were included in the present study. The median age was 61 (53, 67) years, and 811 (70.1%) were male. According to the results of coronary angiography, 317 (27.4%) patients were classified into the CTO group and 840 (72.6%) into the non-CTO group. Baseline characteristics according to CTO status are summarized in Table 1. Patients with CTO were more likely to be male (79.5% vs. 66.5%), current smokers (56.2% vs. 32.4%), have hypertension (65.3% vs. 58.1%), and diabetes mellitus (46.1% vs. 29.2%) than those without CTO (all *p* values < 0.001). Patients with CTO also had higher TyG index, fasting plasma glucose, HbA1c, and creatinine levels, whereas total cholesterol, LDL-C, HDL-C, hemoglobin, platelet count, and LVEF were lower in the CTO group (all *p* values < 0.05). Baseline characteristics according to TyG tertiles are presented in Supplementary Table S1. Patients in the highest TyG tertile (TyG index > 9.139) had a higher prevalence of CTO than those in the lower two tertiles (24.1% vs. 23.9% vs. 34.2%, *p* = 0.001).

### 3.2. Association Between the TyG Index, TyG Tertiles and the Presence of CTO

**Figure 1:**
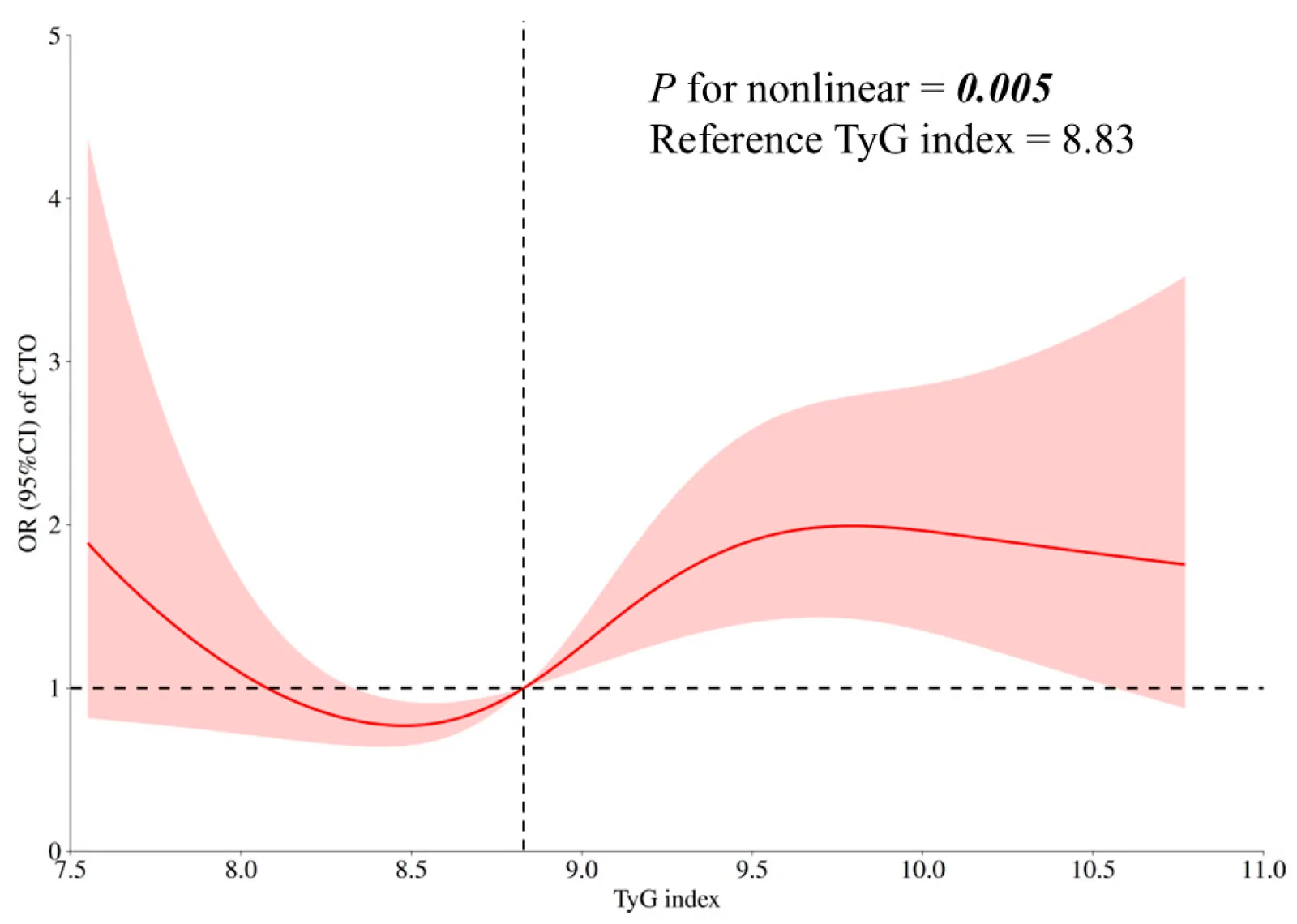
Restricted cubic spline analysis for the OR (95% CI) of CTO. The model was adjusted for current smoking, LDL-C, HDL-C, hemoglobin, and LVEF. The shaded area indicates the 95% confidence interval. CTO, chronic total occlusion; TyG, triglyceride–glucose; LDL-C, low-density lipoprotein cholesterol; HDL-C, high-density lipoprotein cholesterol; LVEF, left ventricular ejection fraction; OR, odds ratio; CI, confidence interval.

Multivariable logistic regression using the Forward: LR method showed that the TyG index was independently associated with the presence of CTO. When analyzed as a continuous variable, each 1-unit increase in the TyG index was associated with higher odds of CTO (OR 1.377, 95% CI 1.082–1.752, *p* = 0.009). Current smoking, LDL-C, HDL-C, hemoglobin, and LVEF remained independently associated with the presence of CTO (all *p* values < 0.001). When the TyG index was further categorized into tertiles, patients in the highest tertile had significantly greater odds of CTO than those in the lowest tertile (OR 1.946, 95% CI 1.272–2.975, *p* = 0.002), whereas no significant association was observed for the middle tertile (OR 1.230, 95% CI 0.805–1.881, *p* = 0.338) (Table 2). Restricted cubic spline curve analysis revealed a nonlinear dose–response relationship (*p* for nonlinear = 0.005) between the TyG index and CTO risk (Figure 1). In the sensitivity analysis further adjusted for diabetes mellitus, the TyG index remained independently associated with the presence of CTO (OR 1.356, 95% CI 1.052–1.747, *p* = 0.018) (Supplementary Table S2). ROC analysis showed that the TyG index alone had limited discriminative ability for CTO (AUC 0.556, 95% CI 0.518–0.595, *p* = 0.003) (Supplementary Table S3).

### 3.3. Subgroup Analysis

**Figure 2:**
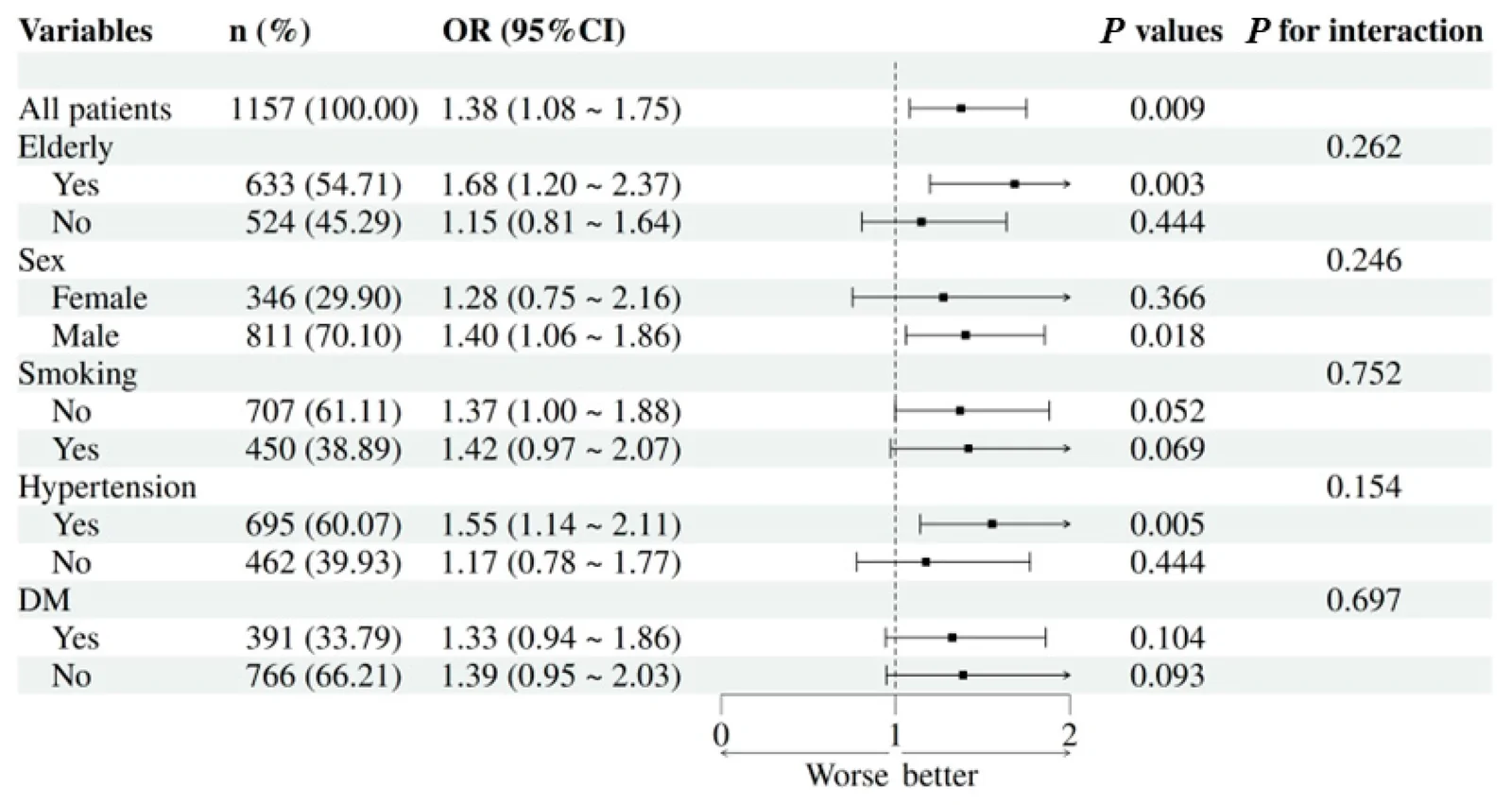
Subgroup analyses for the association of the TyG index and CTO. The subgroup analyses were adjusted for current smoking, LDL-C, HDL-C, hemoglobin, and LVEF, except for the corresponding stratification variable. CTO, chronic total occlusion; TyG, triglyceride–glucose; DM, Diabetes mellitus; LDL-C, low-density lipoprotein cholesterol; HDL-C, high-density lipoprotein cholesterol; LVEF, left ventricular ejection fraction; OR, odds ratio; CI, confidence interval.

Subgroup analyses were performed according to age group, sex, current smoking status, hypertension, and diabetes mellitus (Figure 2). These subgroup models were adjusted for current smoking, LDL-C, HDL-C, hemoglobin, and LVEF, except for the corresponding stratification variable. The association between the TyG index and CTO was directionally consistent across subgroups. Nominally significant associations were observed in elderly participants (OR 1.68, 95% CI 1.20–2.37, *p* = 0.003), men (OR 1.40, 95% CI 1.06–1.86, *p* = 0.018), and patients with hypertension (OR 1.55, 95% CI 1.14–2.11, *p* = 0.005). However, no significant interaction was observed by age group, sex, smoking status, hypertension, or diabetes mellitus (all *p* values for interaction > 0.05). Restricted cubic spline analyses were further performed in the elderly, male, and hypertension subgroups (Supplementary Figure S1). Evidence of nonlinearity was observed only in males (*p* for nonlinearity = 0.045), whereas no significant nonlinearity was found in the elderly (*p* for nonlinearity = 0.277) and hypertension (*p* for nonlinearity = 0.095) subgroups.

## 4. Discussion

In this retrospective angiography-based study, the TyG index was independently associated with the presence of coronary CTO. Patients with CTO had significantly higher TyG index values than those without CTO, and the association remained significant after multivariable adjustment. When the TyG index was analyzed by tertiles, patients in the highest tertile had significantly greater odds of CTO than those in the lowest tertile. Restricted cubic spline analysis suggested a nonlinear dose–response association between the TyG index and the presence of CTO. Importantly, the association between the TyG index and CTO remained significant after additional adjustment for diabetes mellitus, supporting an association beyond baseline diabetes status. In addition, subgroup analyses showed that the association was generally consistent across major clinical subgroups. Although nominally significant associations were observed in elderly participants, men, and patients with hypertension, no significant interaction was detected, indicating no differential effect modification.

The growing evidence indicates that the TyG index reflects not only cardiometabolic risk but also the burden of coronary atherosclerosis. Previous studies have shown that a higher TyG index is associated with an increased risk of CAD, greater lesion severity, and adverse cardiovascular outcomes. A meta-analysis showed that participants in the highest TyG category had a significantly higher risk of CAD during follow-up than those in the lowest category. Similar results were observed when the TyG index was analyzed as a continuous variable [20]. In a study by Saffar Soflaei et al., 2346 participants were classified into five groups based on the number of stenotic coronary vessels. The TyG index increased progressively from healthy individuals to patients with multivessel disease [21]. Wang et al. also reported that a higher TyG index was independently associated with multivessel CAD [11]. Another meta-analysis indicated that CAD patients in the highest TyG category had a significantly higher incidence of major adverse cardiovascular events (MACEs) than those in the lowest category. This relationship was also evident when the TyG index was analyzed as a continuous variable [7]. In CTO populations, previous studies have linked an elevated TyG index to poorer coronary collateralization and adverse long-term outcomes. Gao et al. enrolled 1093 CAD patients with at least one CTO lesion and concluded that a higher TyG index was significantly associated with poorer collateralization [15]. In a prospective cohort study, Song et al. consecutively enrolled 2740 patients with angina and CTO and followed them for a median of 3 years. Patients with a high TyG index had worse clinical outcomes, including all-cause death, cardiovascular death, target-vessel myocardial infarction, ischemia-driven target-vessel revascularization, and stroke, than those with a low TyG index [17]. These studies support the concept that the TyG may be related not only to the presence of CAD, but also to more advanced and complex coronary phenotypes, such as CTO.

Although the mechanism underlying the association between the TyG index and CTO remains unclear, IR, as reflected by the TyG index, may partly explain this relationship. Endothelial dysfunction is a critical initiating factor in the development of atherosclerotic lesions [22,23]. IR contributes to endothelial dysfunction [24,25] and is associated with subclinical vascular injury and coronary atherosclerosis [26]. IR markedly downregulates insulin receptors and insulin receptor-mediated signaling pathways, thereby contributing to impaired endothelial function [27,28,29]. Our previous study demonstrated that an elevated TyG index was independently associated with endothelial dysfunction, providing biological plausibility for a link between the TyG index and CTO [30]. Low-grade chronic inflammation may provide an additional mechanistic link between IR and advanced coronary atherosclerosis. Chronic inflammatory activation contributes to endothelial dysfunction, plaque progression, vascular remodeling, myocardial injury, and adverse cardiovascular outcomes [31,32]. Recent cardiovascular research has increasingly emphasized that inflammation is not limited to acute coronary syndromes but also represents a persistent and incompletely resolved pathophysiological process in chronic cardiovascular disease, including chronic heart failure [31]. The Advanced Lung Cancer Inflammation Index (ALI), a composite index integrating body mass index, albumin, and the neutrophil-to-lymphocyte ratio, has been reported as an independent predictor of all-cause mortality in patients with ST-elevation myocardial infarction undergoing primary PCI [32]. These findings support the potential value of combined inflammatory and nutritional indices in cardiovascular risk assessment. However, in the present retrospective cohort, albumin, neutrophil count, lymphocyte count, high-sensitivity C-reactive protein, and other inflammatory biomarkers were not systematically available. Therefore, ALI and related inflammatory indices could not be reliably calculated or included in the multivariable models. This limitation has been acknowledged and should be addressed in future prospective studies.

More recently, Xiao et al. [18] provided direct evidence for an association between the TyG index and the presence of CTO in a single-center cohort from southwest China. Our study extends these findings by externally validating the association in an independent coronary angiography cohort. Current research on CTO continues to focus on improving patient selection, procedural planning, and revascularization strategies. For example, Acar et al. recently proposed the “Give it time to SOBER up” (GITSU) strategy as a staged approach for CTO-PCI [14]. In this context, simple metabolic markers such as the TyG index may provide complementary information for describing the metabolic profile of patients with CTO. Notably, Xiao et al. reported an apparently linear dose–response pattern between the TyG index and CTO [18], whereas our restricted cubic spline analysis suggested a nonlinear association. Several factors may explain this difference. First, the two studies were conducted in different geographical and clinical settings. Xiao et al. included patients from southwest China, whereas our cohort was derived from an independent northern Chinese angiography-based population. Regional differences in cardiometabolic profiles, dietary patterns, diabetes burden, and clinical treatment strategies may influence the distribution of TyG values and the shape of its association with CTO [33,34]. Second, differences in lipid-lowering therapy may be particularly relevant. In the present study, patients with CTO had markedly lower total cholesterol, LDL-C, and HDL-C levels than those without CTO. This finding may indirectly suggest more intensive lipid-lowering management in the CTO group. Statin therapy can substantially modify lipid profiles and achieved LDL-C levels [35]. Statin therapy may also influence glycemic metabolism and insulin resistance. Previous randomized trial meta-analyses have shown that statins are associated with a small but significant increase in incident diabetes [36]. More recent evidence further suggests that statin therapy can slightly increase HbA1c and HOMA-IR levels [37]. These findings suggest that lipid-lowering therapy and achieved lipid levels may modify the clinical implications of an elevated TyG index.

In subgroup analyses, Xiao et al. showed that the TyG index was associated with a higher likelihood of CTO in males. This is similar to the findings in our study. Likewise, a higher TyG index was independently associated with an increased risk of MACEs in patients with acute myocardial infarction, with stronger prognostic implications in males [38]. These findings suggest that the adverse clinical implications of an elevated TyG index may be more evident in men. However, because all interaction tests were nonsignificant, these subgroup findings should be interpreted as nominally significant associations without differential effect modification.

The heterogeneity of the non-CTO group should also be considered when interpreting our findings. Patients without CTO may include individuals with normal or non-obstructive coronary arteries, as well as those with severe multivessel CAD but no total occlusion. Thus, we cannot determine whether the observed association between the TyG index and CTO is specific to CTO formation or more broadly reflects the relationship between metabolic dysfunction and diffuse coronary atherosclerotic burden. However, detailed angiographic severity metrics, such as the number of diseased vessels, the SYNTAX score, or the Gensini score, were not systematically available in the present database. Therefore, we were unable to define a severe non-CTO CAD subgroup for sensitivity analysis. In addition, although the difference in median TyG values between the CTO and non-CTO groups was small, it remained statistically significant and was supported by multivariable analysis. Therefore, this finding should be interpreted as evidence of an association rather than as a basis for CTO diagnosis. The TyG index may provide additional information on metabolic status in patients undergoing coronary angiography, but its limited discriminative ability indicates that it should not be used alone to identify CTO.

## 5. Limitations

First, this was a single-center cross-sectional study, which cannot demonstrate a causal relationship between the TyG index and the presence of CTO. The TyG index was calculated from a single fasting blood sample obtained at the time of coronary angiography, whereas CTO develops gradually over months or years. Thus, this single measurement may not fully reflect long-term insulin resistance during the actual period of CTO formation. Future longitudinal studies with repeated metabolic measurements are needed to clarify the temporal relationship between the TyG index and CTO development. Second, the study population was enrolled from January 2024 to January 2026, and follow-up data were not available. Accordingly, the association between the TyG index and long-term prognosis could not be evaluated in the present study. Further longitudinal follow-up of this cohort is warranted to clarify the prognostic value of the TyG index in patients with CTO. Third, medication information, including lipid-lowering and glucose-lowering therapies, was not systematically available. These treatments may influence triglyceride, glucose, and lipid levels and may have introduced residual confounding. Fourth, inflammatory markers were incompletely available, and therefore we could not evaluate inflammatory indices such as high-sensitivity C-reactive protein, the neutrophil-to-lymphocyte ratio, or the ALI. Fifth, the non-CTO group was heterogeneous and may have included patients ranging from those with normal coronary arteries to those with severe multivessel CAD without CTO. Therefore, we could not determine whether the TyG index was specifically associated with CTO formation or more broadly reflected diffuse coronary atherosclerotic burden.

## 6. Conclusions

An elevated TyG index was independently associated with the presence of CTO, and this association remained significant after further adjustment for diabetes mellitus. Restricted cubic spline analysis suggested a nonlinear dose–response association between the TyG index and CTO. Subgroup analyses showed that the association was generally consistent across major clinical subgroups, with no significant interaction observed. These findings suggest that the TyG index may serve as an adjunctive metabolic marker for characterizing patients with CTO.

## Figures and Tables

**Table 1:** Baseline characteristics according to CTO status.

Variables	Total (n = 1157)	Non-CTO (n = 840)	CTO (n = 317)	*p* Value
Age, years	61 (53, 67)	61 (53, 67)	61 (54, 68)	0.182
Male, n (%)	811 (70.1)	559 (66.5)	252 (79.5)	<0.001
BMI, kg/m^2^	25.61 (23.36, 27.93)	25.50 (23.36, 27.82)	25.69 (23.44, 28.28)	0.608
Current smoking, n (%)	450 (38.9)	272 (32.4)	178 (56.2)	<0.001
Hypertension, n (%)	695 (60.1)	488 (58.1)	207 (65.3)	0.030
Diabetes mellitus, n (%)	391 (33.8)	245 (29.2)	146 (46.1)	<0.001
Laboratory results				
TyG index	8.83 (8.42, 9.34)	8.79 (8.41, 9.26)	8.98 (8.46, 9.45)	0.003
Fasting plasma glucose, mmol/L	5.41 (4.83, 6.70)	5.25 (4.80, 6.33)	6.10 (4.96, 7.68)	<0.001
Triglycerides, mmol/L	1.50 (1.09, 2.15)	1.48 (1.08, 2.15)	1.59 (1.11, 2.12)	0.412
HbA1c, %	6.20 (5.60, 6.40)	6.05 (5.50, 6.20)	6.30 (5.90, 6.80)	<0.001
Creatinine, μmol/L	69.00 (58.00, 79.00)	68.00 (57.00, 77.00)	72.80 (62.20, 84.20)	<0.001
Uric acid, μmol/L	337 (278, 398)	339 (284, 398)	331 (271, 394)	0.132
Total cholesterol, mmol/L	3.87 (3.16, 4.66)	4.12 (3.53, 4.90)	3.11 (2.63, 3.66)	<0.001
LDL-C, mmol/L	2.23 (1.64, 2.88)	2.45 (1.98, 3.04)	1.53 (1.16, 1.96)	<0.001
HDL-C, mmol/L	1.04 (0.87, 1.25)	1.10 (0.93, 1.34)	0.89 (0.76, 1.02)	<0.001
White blood cell count, ×10^9^/L	6.34 (5.27, 7.71)	6.27 (5.24, 7.61)	6.54 (5.39, 8.00)	0.089
Hemoglobin, g/L	141 (130, 150)	142 (132, 152)	137 (127, 147)	<0.001
Platelet count, ×10^9^/L	211 (179, 249)	212 (181, 252)	204 (174, 238)	0.005
Transthoracic Echocardiography				
LVEF, %	65 (60, 68)	66 (61, 69)	63 (54, 66)	<0.001

CTO, chronic total occlusion; BMI, body mass index; TyG, triglyceride–glucose; LDL-C, low-density lipoprotein cholesterol; HDL-C, high-density lipoprotein cholesterol; LVEF, left ventricular ejection fraction.

**Table 2:** Multivariable logistic regression analyses for the presence of CTO.

Variables	OR	(95% CI)	*p* Values
Model 1: including the TyG index			
Current smoking	2.150	(1.520–3.042)	<0.001
LDL-C	0.226	(0.175–0.293)	<0.001
HDL-C	0.154	(0.074–0.319)	<0.001
Hemoglobin	0.977	(0.967–0.988)	<0.001
LVEF	0.943	(0.926–0.961)	<0.001
TyG index	1.377	(1.082–1.752)	0.009
Model 2: including the TyG tertiles			
Current smoking	2.146	(1.516–3.039)	<0.001
LDL-C	0.219	(0.168–0.285)	<0.001
HDL-C	0.156	(0.075–0.324)	<0.001
Hemoglobin	0.977	(0.966–0.988)	<0.001
LVEF	0.944	(0.927–0.962)	<0.001
TyG tertiles			
Medium vs. Low	1.230	(0.805–1.881)	0.338
High vs. Low	1.946	(1.272–2.975)	0.002

CTO, chronic total occlusion; TyG, triglyceride–glucose; LDL-C, low-density lipoprotein cholesterol; HDL-C, high-density lipoprotein cholesterol; LVEF, left ventricular ejection fraction; OR, odds ratio; CI, confidence interval.

## Data Availability

The data supporting the findings of this study are not publicly available due to patient privacy and ethical restrictions. The dataset contains sensitive clinical information derived from hospital records and coronary angiography, and public sharing was not included in the ethics approval framework for this study. De-identified data can be made available from the corresponding author upon reasonable request, subject to institutional approval and applicable ethical requirements.

## Supplementary Materials

The following supporting information can be downloaded at https://www.mdpi.com/article/10.3390/jcdd13060275/s1, Table S1: Baseline characteristics according to the TyG tertiles. Table S2: Sensitivity analysis adjusting for diabetes mellitus. Table S3: ROC curve analysis for CTO discrimination. Figure S1: Subgroup-specific restricted cubic spline analyses for the OR (95% CI) of CTO in the elderly participants, males, and patients with hypertension. (A), the association in the elderly; (B), the association in males; (C), the association in patients with hypertension. These models were adjusted for current smoking, LDL-C, HDL-C, hemoglobin, and LVEF. The shaded area indicates the 95% confidence interval. CTO, chronic total occlusion; TyG, triglyceride-glucose; LDL-C, low-density lipoprotein cholesterol; HDL-C, high-density lipoprotein cholesterol; LVEF, left ventricular ejection fraction; OR, odds ratio; CI, confidence interval.
