# Supplementary material for: Association of Triglyceride–Glucose Index and Coronary Chronic Total Occlusion in Patients Undergoing Coronary Angiography: A Retrospective Study
Source: J Cardiovasc Dev Dis. 2026 Jun 17;13(6):275. doi: 10.3390/jcdd13060275 (PMC13300926; doi:10.3390/jcdd13060275)
Supplement: Supplementary file 1 [file jcdd-13-00275-s001.zip › jcdd-4328688-supplementary.pdf]

**Table S1 Baseline characteristics according to the TyG tertiles**

| Variables | Low tertile (n=386) (TyG < 8.560) | Medium tertile (n=385) (8.560 ≤ TyG ≤ 9.139) | High tertile (n=386) (TyG > 9.139) | P value |
|---|---|---|---|---|
| Age, years | 61 (54, 68) | 61 (54, 67) | 60 (53, 67) | 0.230 |
| Male, n (%) | 258 (66.8) | 270 (70.1) | 283 (73.3) | 0.145 |
| BMI, kg/m² | 24.37 (22.49, 26.70) | 25.71 (23.74, 28.09) | 26.46 (24.47, 28.81) | ***<0.001*** |
| Current smoking, n (%) | 125 (32.4) | 147 (38.2) | 178 (46.1) | ***<0.001*** |
| Hypertension, n (%) | 211 (54.7) | 239 (62.1) | 245 (63.5) | ***0.027*** |
| Diabetes mellitus, n (%) | 78 (20.2) | 106 (27.5) | 207 (53.6) | ***<0.001*** |
| ***Laboratory results*** | | | | |
| **TyG index** | 8.26 (8.04, 8.42) | 8.83 (8.71, 8.98) | 9.52 (9.34, 9.90) | ***<0.001*** |
| Fasting plasma glucose, mmol/L | 4.92 (4.56, 5.37) | 5.34 (4.89, 6.15) | 7.08 (5.70, 9.59) | ***<0.001*** |
| Triglycerides, mmol/L | 0.95 (0.79, 1.14) | 1.55 (1.39, 1.78) | 2.56 (1.99, 3.31) | ***<0.001*** |
| HbA1c, % | 5.90 (5.50, 6.20) | 6.10 (5.60, 6.30) | 6.30 (5.90, 7.40) | ***<0.001*** |
| Creatinine, μmol/L | 67.00 (57.00, 76.50) | 69.80 (59.00, 81.00) | 70.00 (59.00, 80.00) | ***0.029*** |
| Uric acid, μmol/L | 310 (266, 370) | 344 (285, 405) | 359 (298, 418) | ***<0.001*** |
| Total cholesterol, mmol/L | 3.55 (2.92, 4.33) | 3.91 (3.23, 4.66) | 4.10 (3.35, 4.88) | ***<0.001*** |
| LDL-C, mmol/L | 2.00 (1.51, 2.70) | 2.39 (1.77, 3.00) | 2.29 (1.70, 2.97) | ***<0.001*** |
| HDL-C, mmol/L | 1.18 (0.96, 1.49) | 1.02 (0.87, 1.25) | 0.95 (0.82, 1.09) | ***<0.001*** |
| White blood cell count, $\times 10^9$/L | 5.98 (4.79, 7.00) | 6.40 (5.44, 7.86) | 6.68 (5.56, 8.23) | ***<0.001*** |
| Hemoglobin, g/L | 139 (128, 148) | 142 (132, 150) | 143 (132, 154) | ***<0.001*** |
| Platelet count, $\times 10^9$/L | 205 (175, 241) | 212 (181, 248) | 214 (182, 252) | ***0.037*** |
| ***Transthoracic Echocardiography*** | | | | |
| LVEF, % | 66 (60, 68) | 66 (60, 69) | 64 (59, 68) | ***0.020*** |
| ***Coronary angiography*** | | | | |
| CTO, n (%) | 93 (24.1) | 92 (23.9) | 132 (34.2) | ***0.001*** |

BMI, body mass index; TyG, triglyceride-glucose; LDL-C, low density lipoprotein cholesterol; HDL-C, high density lipoprotein cholesterol; LVEF, left ventricular ejection fraction; CTO, chronic total occlusion;.

**Table S2 Sensitivity analysis adjusting for diabetes mellitus**

| Variables | OR | (95%CI) | P values |
|---|---|---|---|
| Current smoking | 2.149 | (1.518–3.044) | **<0.001** |
| LDL-C | 0.226 | (0.175–0.293) | **<0.001** |
| HDL-C | 0.154 | (0.074–0.320) | **<0.001** |
| Hemoglobin | 0.977 | (0.967–0.988) | **<0.001** |
| LVEF | 0.943 | (0.926–0.961) | **<0.001** |
| Diabetes mellitus | 1.072 | (0.757–1.518) | 0.695 |
| TyG index | 1.356 | (1.052–1.747) | **0.018** |

LDL-C, low-density lipoprotein cholesterol; HDL-C, high-density lipoprotein cholesterol; LVEF, left ventricular ejection fraction; TyG, triglyceride-glucose; OR, odds ratio; CI, confidence interval.

## Table S3 ROC curve analysis for CTO discrimination

| Model | AUC | (95%CI) | P value |
|---|---|---|---|
| TyG index | 0.556 | (0.518–0.595) | **0.003** |

ROC, receiver operating characteristic; CTO, chronic total occlusion; AUC, area under the curve; CI, confidence interval; TyG, triglyceride-glucose.

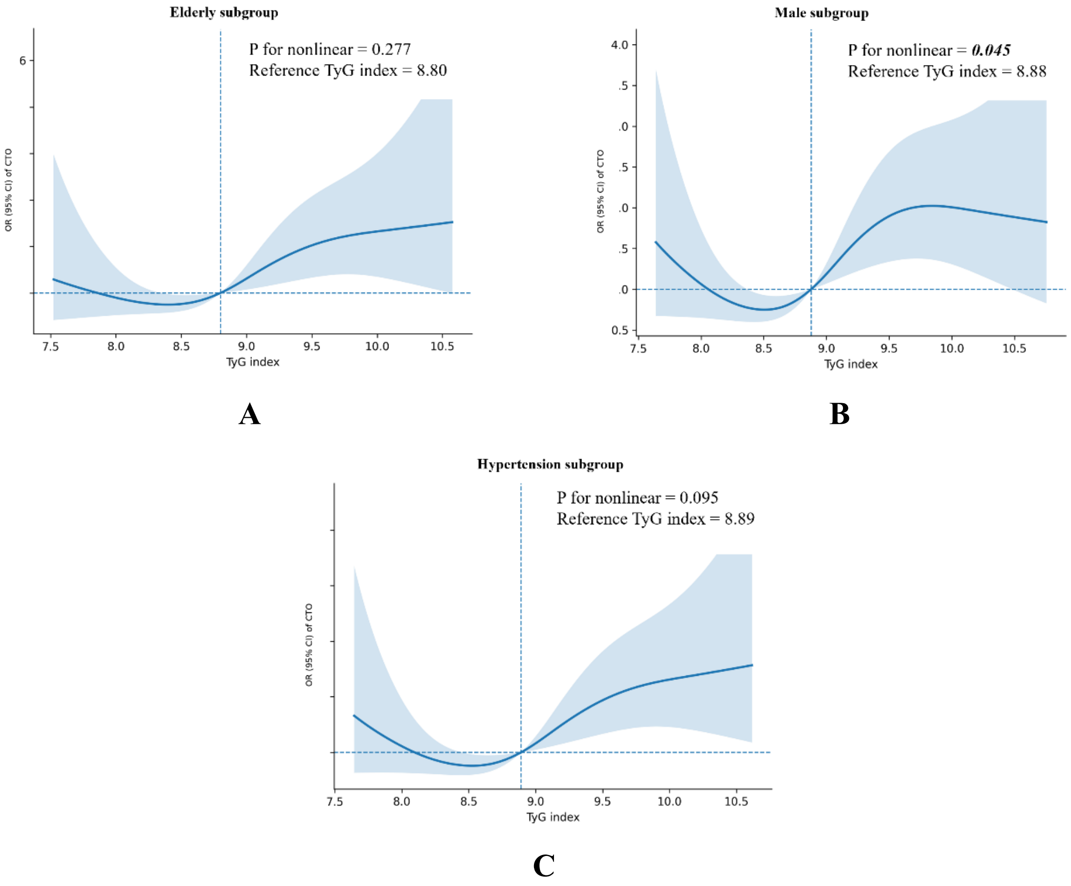


**Figure S1. Subgroup-specific restricted cubic spline analyses for the OR (95%CI) of CTO in the elderly participants, males, and patients with hypertension**. A, the association in the elderly; B, the association in males; C, the association in patients with hypertension. These models were adjusted for current smoking, LDL-C, HDL-C, hemoglobin, and LVEF. The shaded area indicates the 95% confidence interval. CTO, chronic total occlusion; TyG, triglyceride-glucose; LDL-C, low-density lipoprotein cholesterol; HDL-C, high-density lipoprotein cholesterol; LVEF, left ventricular ejection fraction; OR, odds ratio; CI, confidence interval.
